# Integrative Taxonomy Reveals a New Species of the *Eisenia andrei/fetida* Complex (Clitellata: Lumbricidae) From the Guizhou Plateau, China

**DOI:** 10.1002/ece3.73670

**Published:** 2026-05-11

**Authors:** Tingchang Liu, Rongchuan Xiong, Hong Chen, Jianhong Li, Tingting Ji

**Affiliations:** ^1^ School of Biological Science and Technology Liupanshui Normal University Liupanshui China

**Keywords:** cryptic species, earthworm, *Eisenia*, Guizhou Plateau, integrative taxonomy

## Abstract

The *Eisenia andrei/fetida* complex represents one of the most widely distributed and taxonomically challenging groups of earthworms, exhibiting high cryptic diversity and complex species boundaries. Here, we describe a new species, *Eisenia zhongshanensis* sp. nov., from Wangjiazhai Town, Zhongshan District, Liupanshui City, located on the Guizhou Plateau, China. Species delimitation was performed using an integrative taxonomic approach combining morphological examination and molecular phylogenetic analyses based on two molecular markers: the mitochondrial cytochrome *c* oxidase subunit I (*COI*) gene and the nuclear 28S ribosomal RNA (*28S* rRNA) gene. Phylogenetic analyses revealed that the new species belongs to the *Eisenia fetida* complex but forms a highly supported independent lineage (posterior probability ≥ 0.95; bootstrap value ≥ 70%). The new species is distinguished from all known congeners by its rhombus‐shaped spermathecae, three pairs of spermathecal pores in 6/7/8/9, and unique tanylobous prostomium. The genetic distances (K2P) between the new species and its closest relatives ranged from 12.3% to 15.0% for COI, and 1.7% to 2.1% for 28S rRNA. This discovery highlights the underestimated biodiversity of the *Eisenia* complex in high‐altitude regions of southwestern China and underscores the necessity of applying integrative taxonomic approaches to resolve cryptic diversity in lumbricid earthworms.

## Graphical abstract

We used mitochondrial COI genes and nuclear gene 28S rRNA genes to investigate the phylogenetic relationships and evolutionary history of an unknown earthworm genus collected in Wangjiazhai Town, Zhongshan District, Liupanshui City. The results of molecular analyses supported it belongs to an *Eisenia andrei* /*fetida* complex. Furthermore, the dissected results show the spermathecal of *Eisenia zhongshanensis* sp. nov. is rhombus‐shaped, and its intestinal caeca horn‐shaped, which differ from that of the most recent species genus *Eisenia fetida* (Savigny, 1826) and *Eisenia andrei* (Bouché, 1972).

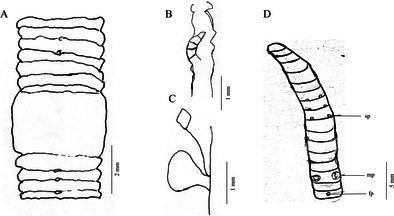

## 1 Introduction

Earthworms (Annelida: Clitellata) are predominantly terrestrial invertebrates comprising approximately 5500 described species (Blakemore et al. 2006). As ubiquitous ecosystem engineers, they fundamentally influence soil structure, organic matter mineralization, and nutrient cycling (Michael et al. 2025; Ortíz‐Gamino et al. 2020), while serving as crucial decomposers and food resources for soil fauna (Seesamut et al. 2019). Despite their ecological importance, earthworm taxonomy has been historically constrained by the scarcity of taxonomically informative morphological characters and high levels of homoplasy, leading to the prevalence of cryptic species complexes (Marchán et al. 2022; Chang and James 2011).

The genus *Eisenia* (Lumbricidae) includes several extensively studied species, notably *Eisenia fetida* (Savigny, 1826) and *Eisenia andrei* (Bouché, 1972), which serve as standard model organisms in ecotoxicology worldwide (Dominguez and Aira 2022; Römbke et al. 2016). Historically, these two species were considered morphotypes or subspecies (*E. foetida fetida* and *E. foetida unicolor*) until biochemical and molecular evidence demonstrated their reproductive isolation (Jaenike 1982; André 1963). Phylogenetic analyses using mitochondrial *COI* and nuclear *28S* rRNA sequences have confirmed that they represent separate biological species, with *COI* genetic distances reaching 15.0% (Pérez‐Losada et al. 2005; Pop et al. 2007). However, recent DNA barcoding studies have revealed that the *E. fetida/andrei* complex harbors substantial cryptic diversity; *E. fetida* alone comprises at least two cryptic lineages, while *E. andrei* contains six deeply diverged mitochondrial lineages (Römbke et al. 2016; Dhakane and Shinde 2020). Clarifying these hidden lineages and their formation mechanisms relies on more comprehensive population sampling through morphological and molecular studies, especially in remote areas that are far from the type locality and have special habitats, to avoid human interference.

Liupanshui City (25°19′44″–26°55′33″N, 104°18′20″–105°42′50″E) is situated on the Guizhou Plateau in southwestern China, characterized by a mean annual temperature below 13°C and unique karst topography (Ao 2016; Sun et al. 2025). The particular geographical and climatic conditions of this high‐altitude region create distinctive habitats that may foster speciation and endemism (Wang et al. 2024; Rillig et al. 2019). However, the earthworm fauna of this area remains poorly documented. In the present study, we report the discovery of an unknown earthworm species belonging to the *E. fetida/andrei* complex collected from farmland in Wangjiazhai Village. Through integrative taxonomic analysis, we herein describe this taxon as a new species, *Eisenia zhongshanensis* sp. nov., contributing to the understanding of earthworm biodiversity in the Guizhou Plateau.

## 2 Materials and Methods

### 2.1 Specimen Collection and Preservation

Earthworms were collected from farmland (26°34′–26°45′N, 104°50′–105°00′ E) in Wangjiazhai Town, Liupanshui City, Guizhou Province, China, using digging and hand sorting methods. Specimens were rinsed with distilled water, fixed in 95% ethanol, then stored at −20°C. Holotype (WJZ‐1) and six paratypes (WJZ‐2–7) are deposited in the School of Biological Science and Technology, Liupanshui Normal University.

### 2.2 Molecular Marker Selection Rationale

In this study, we employed two complementary molecular markers with distinct evolutionary rates: the mitochondrial *COI* gene and the nuclear *28S* rRNA gene. The selection was based on their established utility in resolving different taxonomic levels in earthworm systematics (Chang et al. 2008; Huang et al. 2007).

The *COI* gene exhibits rapid evolutionary rates (approximately 4.8% per million years in earthworms) and high genetic variability at the species level, making it ideal for DNA barcoding and species‐level identification (Hebert et al. 2003). However, due to its fast evolution, *COI* suffers from nucleotide saturation and limited resolution for deeper phylogenetic relationships. In contrast, the nuclear *28S* rRNA gene evolves more slowly and possesses substantial slowly evolving sites that provide stable phylogenetic signals for resolving generic relationships (James and Davidson 2012; Halanych and Janosik 2006). Previous studies have demonstrated that *28S* rRNA is superior to mitochondrial markers for inferring higher‐level relationships among lumbricid earthworms, whereas *COI* provides high resolution for distinguishing closely related species (Mardulyn and Whitfield 1999; Kim et al. 2014).

The combined use of these markers allows for cross‐validation of species boundaries and mitigates the limitations of single‐gene analyses, particularly in detecting potential mitochondrial introgression or incomplete lineage sorting that may confound species delimitation in the *Eisenia* complex (Martinsson and Erseus 2021; Martinsson and Erseus 2017).

### 2.3 Molecular Protocols and Phylogenetic Analysis

Total RNA was extracted from tail tissue samples, and transcriptome sequencing libraries were constructed and sequenced on the Illumina NovaSeq 6000 platform. Raw reads were quality‐filtered, and high‐quality full‐length transcripts (Unigenes) were obtained through de novo assembly. Target gene sequences were extracted using local BLAST searches against a constructed reference database containing mitochondrial *COI* and nuclear *28S* rRNA sequences from Annelida. Then the retrieved *COI* and nuclear *28S* rRNA sequences were uploaded to GenBank for BLASTn, yielding 250 and 100 homologous DNA sequences, respectively. The target sequences and their homologous DNA sequences were combined and aligned using MUSCLE (Edgar 2004) respectively for further phylogenetic analyses.

Phylogenetic analyses were performed using three approaches: (1) Neighbor‐Joining (NJ) trees based on p‐distances with pairwise deletion; (2) Maximum Likelihood (ML) using IQ‐TREE under the optimal substitution model selected by ModelFinder (GTR + I + G, G = 0.885, *I* = 0.605) (Kalyaanamoorthy et al. 2017); and (3) Bayesian Inference (BI) using MrBayes 3.2 (Ronquist et al. 2012). Branch support was assessed using bootstrap values (≥ 70% considered strong support for NJ and ML) and posterior probabilities (≥ 0.95 considered strong support for BI) (Hillis and Bull 1993).

### 2.4 Morphological Examination

Specimens were dissected and examined under a stereo microscope following standard earthworm taxonomic protocols (Blakemore 2008). External characters recorded included body length, width, segment number, prostomium shape, pigmentation pattern, and positions of clitellum and genital pores. Internal characters examined included septal thickness, gizzard, hearts, seminal vesicles, and spermathecae shape. Illustrations were prepared for diagnostic characters.

### 2.5 Species Delimitation Criteria

Species boundaries were determined using an integrative taxonomic approach (Padial et al. 2010; Schlick‐Steiner et al. 2010) combining morphological distinctiveness and molecular divergence. Morphological criteria followed the general lineage species concept (de Queiroz 1998; de Queiroz 2007), focusing on fixed diagnostic differences in reproductive structures. Molecular criteria followed the phylogenetic species concept, with reciprocal monophyly and genetic distance thresholds (*COI* K2P > 10% for congeneric species) serving as primary evidence (Decaëns et al. 2013; Latif et al. 2017).

## 3 Results

### 3.1 Molecular Phylogenetic Analyses

**FIGURE 1:**
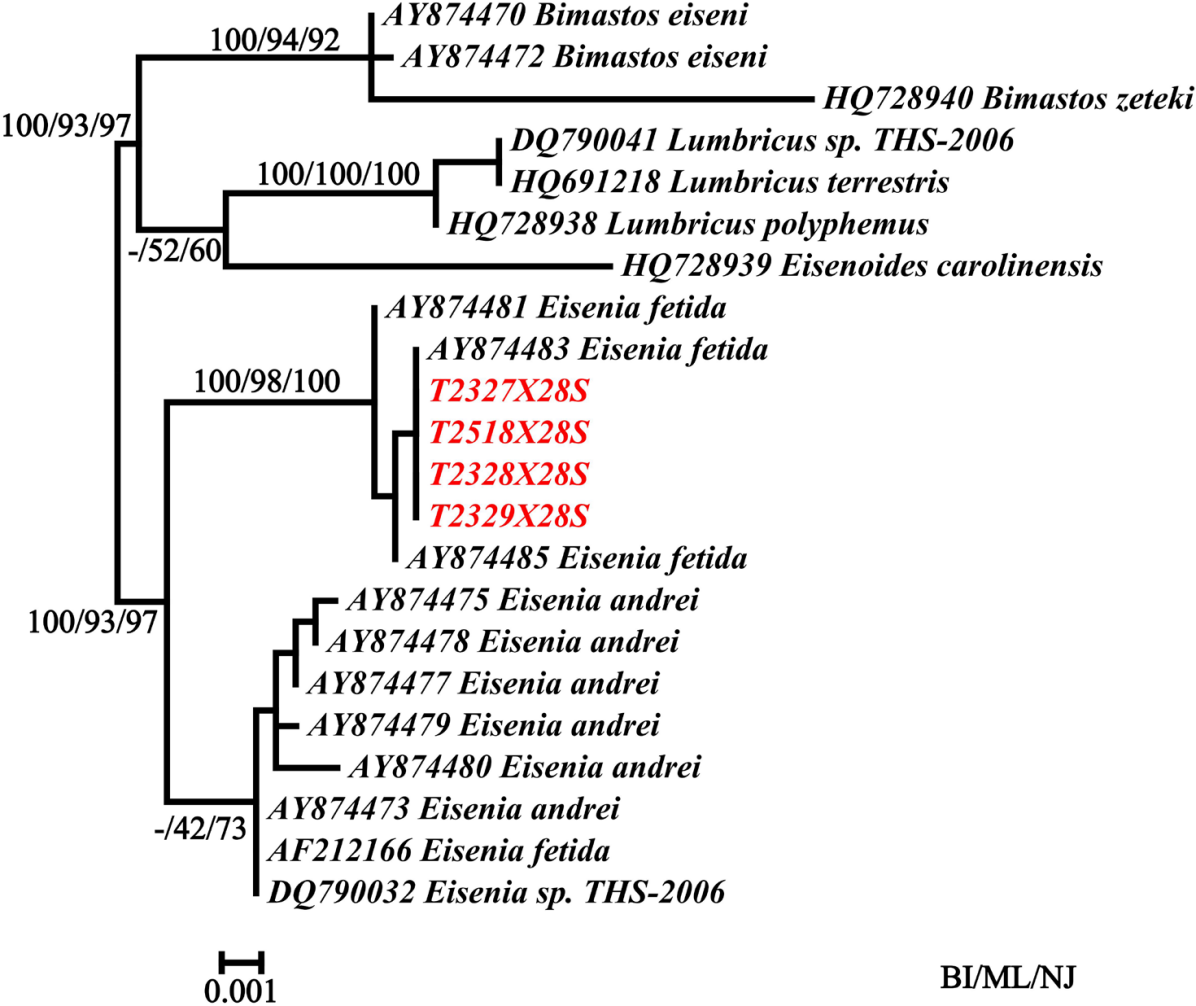
Phylogenetic analysis of *Eisenia zhongshanensis* sp. nov. by *28S* gene.

Based on the *28S* rRNA phylogeny, the specimens from Liupanshui clearly clustered within the *Eisenia fetida/andrei* complex, showing close affinity to both *E. fetida* and *E. andrei* (Figure 1), consistent with previous phylogenetic hypotheses (Pokryszko et al. 2018). To further resolve species boundaries, we downloaded all available *COI* sequences of *E. fetida* and *E. andrei* from GenBank, merged them with our novel sequences, and constructed haplotype‐based phylogenies.

**FIGURE 2:**
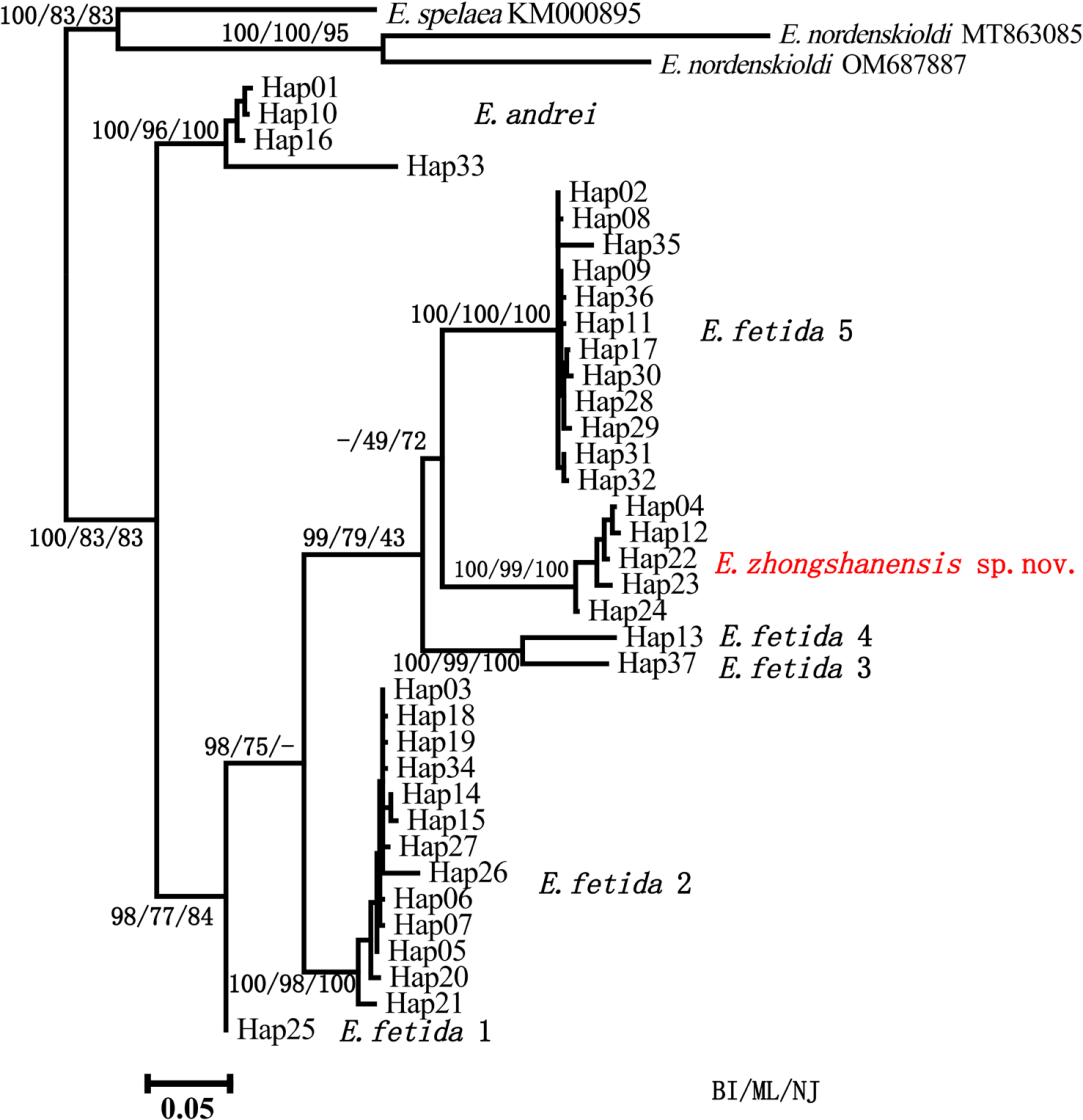
Phylogenetic analysis of *Eisenia zhongshanensis* sp. nov. by *COI* gene.

The *COI* phylogeny revealed that the *Eisenia fetida* complex comprises at least six distinct, well‐supported clades ( *E. fetida* 1–6) plus *E. andrei* (Figure 2). The sequences of specimens from Liupanshui (Hap04) clustered with the sequences from some Europe (Hap22‐24) specimens to form an independent lineage ( *E. fetida* 6) with high statistical support (bootstrap ≥ 70%, posterior probability ≥ 0.95), exhibiting substantial genetic divergence from other clades. Genetic distance analysis based on K2P revealed that the new lineage diverged from *E. fetida* sensu stricto by 12.3%–14.5% and from *E. andrei* by 13.8%–15.0% for *COI*, and by 1.7%–2.1% for *28S* rRNA. These distances exceed the standard thresholds for species delimitation in lumbricid earthworms (typically 10%–13% for *COI*) (Kvist 2013). The above results are consistent with that the obtained specimens are most similar to the genus *Eisenia* (Malm, 1877) (Table 1).

**TABLE 1:** Holotype morphological characteristics of *Eisenia zhongshanensis* sp. nov. and *Eisenia malm*,1877.

Species	Mouth	Clitellum	Male pore	Spermathecal pore	Gizzard	Heart
*Eisenia zhongshanensis* sp. nov.	Tanylobous prostomium	Saddle‐shaped	XV	3 pairs	Longer than a segment	Start from VII segment
*Eisenia malm*,1877	Epilobous, proepilobous, tanylobous	Saddle‐shaped	XV	2–3 pairs	Longer than a segment	Start from VII segment

### 3.2 Morphological Diagnosis: *Eisenia zhongshanensis* sp. Nov

#### 3.2.1 Material Examined

**FIGURE 3:**
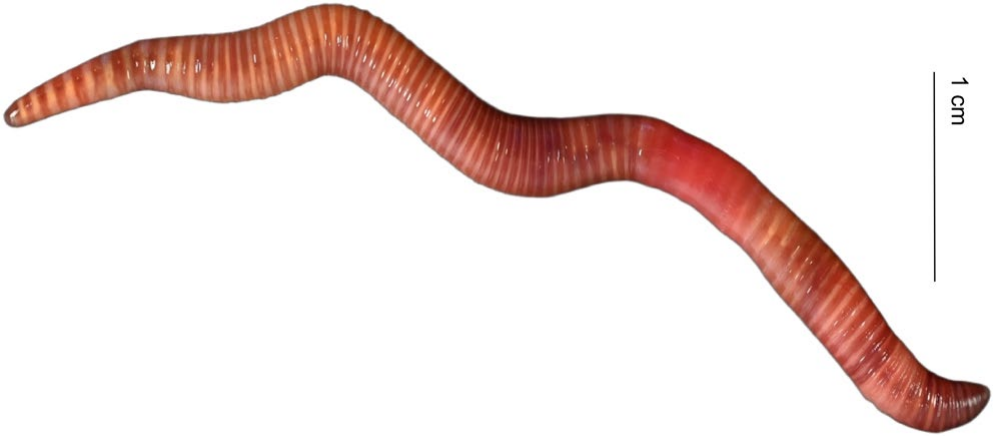
External view of body of *Eisenia zhongshanensis* sp. nov. holotype.

Holotype: WJZ‐1, single complete mature specimen from Wangjiazhai Town (26°34′–26°45′ N, 104°50′–105°00′ E), Liupanshui City, Guizhou Province, China. Collected by R. Xiong and T. Liu (Figure 3). Six paratypes (WJZ‐2–7) collected from the same locality. Holotype and paratypes deposited in the School of Biological Science and Technology, Liupanshui Normal University.

#### 3.2.2 Diagnosis

**FIGURE 4:**
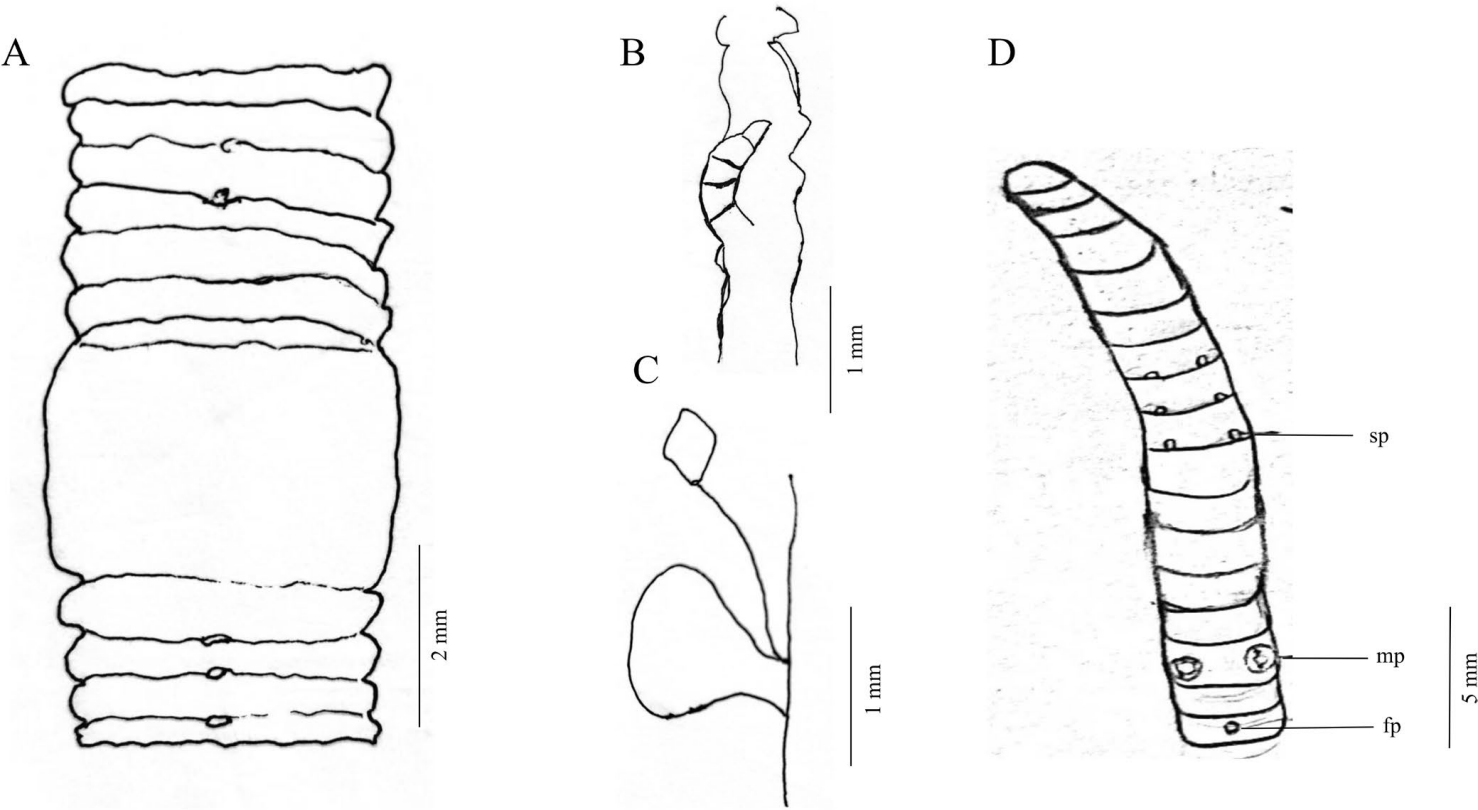
*Eisenia zhongshanensis* sp. nov. (A, D) ventral view showing spermathecal pores, and male pores (B) intestine (C) spermatheca and accessory glands. fp, female pores; mp, male pores; Sp., spermathecal pores.

*Eisenia zhongshanensis* sp. nov. is distinguished from all congeners by the following combination of characters: (1) tanylobous prostomium (vs. proepilobous in *E. fetida* ) (Table 2); (2) rhombus‐shaped spermathecae with heart‐shaped ampullae (vs. heart‐shaped or cylindrical spermathecae in related species) (Figure 4C); (3) three pairs of spermathecal pores in intersegments 6/7/8/9 (vs. two pairs in *E. fetida* ) (Table 2); (4) male pores surrounded by three papillae within a cuticular fold (Figure 4D); (5) gizzard elongated, bucket‐shaped, occupying segments XV–XVIII (Figure 4B).

**TABLE 2:** Holotype morphological characteristics of *Eisenia zhongshanensis* sp. nov., *Eisenia fetida* (Savigny, 1826) and *Eisenia andrei* (Bouché, 1972).

Species	Mouth	Clitellum	Body length/mm	Diameter/mm	Segment number	Seta	Male pore	Spermathecal pore	Body pigment
*Eisenia fetida* (Savigny,1826)	Proepilobous	VI–VIII, saddle‐shaped	60–120	3–6	80–120	dd = 1/2 body circumference in leading end, dd < 1/2 body circumference in trailintg end	Has a large glandular mastoid	2 pairs	Redbrown strip
*Eisenia andrei* (Bouché, 1972)		VI–VIII, saddle‐shaped	60–120	3–6	80–120				Uniformly red
*Eisenia zhongshanensis* sp. nov.	Tanylobous prostomium	XXVI–XXXII, saddle‐shaped	65–113	3	76–98	dd = 1/2 body circumference	Surrounded by skin folds along with three papillae	3 pairs	Redbrown strip
*Eisenia nordenskioldi* nordenskioldi (Eisen, 1878)	Proepilobous	XXVI, XVII–XXXII, XXXIII, saddle‐shaped	50–110	3–6	124–165	aa > bc, dd < 1/2 body circumference	XV	2 pairs	Light‐yellow, no stripes

#### 3.2.3 External Characters

Length 65–113 mm, diameter 3 mm at clitellum, 76–98 segments. Prostomium tanylobous. Pigmentation light brown dorsally, yellowish‐brown ventrally, with dark brown annuli and distinct purplish‐brown dorsal midline. First dorsal pore in segment 9. Clitellum saddle‐shaped, pinkish, smooth, prominent, occupying segments XXVI–XXXII, lacking dorsal pores and setae (Figure 4A). Setae paired, opposite, formula aa:ab:bc:cd:dd = 1:1:1:6. Male pores paired in segment XV, each on pad‐like protrusion surrounded by three papillae within cuticular fold. Spermathecal pores three pairs in 6/7/8/9.

#### 3.2.4 Internal Characters

Septa thick anterior to segment 11, especially thickened at septum 11. Gizzard elongated, bucket‐shaped, in XV–XVIII. Intestine gradually enlarged from segment XV. Pseudohearts three or four pairs in VII–VIII. Seminal vesicles four pairs in IX and XII. Spermathecae yellow, dot‐shaped, well‐developed on seminal vesicles, rhombus‐shaped (diagnostic) (Figure 4C), left and right connected on ventral margins. Ampullae heart‐shaped, ducts approximately 1/2 ampulla length. Accessory glands milky‐white, flower‐shaped, in segment X. Prostates small, underdeveloped.

#### 3.2.5 Ecology

Found in farmland soils rich in organic matter.

#### 3.2.6 Etymology

The specific epithet *zhongshanensis* refers to Zhongshan District, Liupanshui City, the type locality.

## 4 Discussion

### 4.1 Taxonomic Status and Cryptic Diversity of the *E. fetida/andrei* Complex

The *E. fetida/andrei* complex has long been recognized as a taxonomically challenging group characterized by morphological similarity yet substantial genetic diversity (Pérez‐Losada et al. 2005; Römbke et al. 2016). Our phylogenetic analyses confirm that the new species from Liupanshui represents a distinct evolutionary lineage within this complex. The genetic distances between *E. zhongshanensis* sp. nov. and its closest relatives exceed the standard barcoding thresholds for earthworm species delimitation (10%–13% for *COI*), strongly supporting its status as a separate species (Hebert et al. 2003). Furthermore, the reciprocal monophyly observed in both mitochondrial and nuclear gene trees provides robust evidence for reproductive isolation and independent evolutionary history (de Queiroz 2007).

### 4.2 Evidence for Species Status of *Eisenia zhongshanensis* sp. Nov

The description of *E. zhongshanensis* sp. nov. as a new species is supported by multiple lines of evidence following the integrative taxonomic approach (Padial et al. 2010; Schlick‐Steiner et al. 2010):

First, *diagnostic morphological autapomorphies*: The rhombus‐shaped spermathecae constitute a unique morphological character state not observed in any described *Eisenia* species. The presence of three pairs of spermathecal pores (vs. two pairs in *E. fetida* and *E. andrei* ) and the tanylobous prostomium further distinguish the new species.

Second, *molecular divergence and phylogenetic independence*: The new species exhibits substantial genetic differentiation from all known congeners (*COI* K2P > 12%; *28S* > 1.7%), exceeding species‐level thresholds. Both NJ, ML, and BI analyses recover *E. zhongshanensis* sp. nov. as a highly supported independent clade, indicating long‐term reproductive isolation.

Third, *geographic isolation*: The Guizhou Plateau represents a distinct biogeographic region with unique climatic and edaphic conditions that may have promoted allopatric speciation. The restricted distribution of the new species to high‐altitude farmland habitats in Liupanshui suggests local adaptation and limited dispersal, consistent with the low vagility characteristic of lumbricid earthworms (Fernández et al. 2012).

Fourth, *absence of intermediates*: No morphologically intermediate specimens or evidence of hybridization (e.g., mitochondrial‐nuclear discordance) were detected in the sampled population, supporting species integrity.

The congruence between morphological novelty and molecular divergence provides compelling evidence that *E. zhongshanensis* represents a separately evolving metapopulation lineage (de Queiroz 1998), fulfilling the criteria for species recognition under the general lineage species concept.

### 4.3 Methodological Considerations

The application of both fast‐evolving (*COI*) and slow‐evolving (*28S* rRNA) molecular markers proved essential for resolving the systematic position of the new species. While *COI* provided sufficient resolution to distinguish cryptic lineages within the *E. fetida* complex, *28S* rRNA confirmed the deeper phylogenetic placement within *Eisenia* and detected no evidence of mitochondrial introgression (James and Davidson 2012; Mardulyn and Whitfield 1999). This dual‐marker approach aligns with recent recommendations for earthworm integrative taxonomy, emphasizing the necessity of combining mitochondrial barcodes with nuclear phylogenetic markers to robustly delimit species boundaries (Liu et al. 2025).

## 5 Conclusions

This study describes a new species of the *Eisenia andrei/fetida* complex, *Eisenia zhongshanensis* sp. nov., from the Guizhou Plateau, China, based on an integrative taxonomic approach combining morphological examination and molecular phylogenetic analysis. The new species is diagnosed by unique rhombus‐shaped spermathecae, three pairs of spermathecal pores, and a tanylobous prostomium and is supported by substantial molecular divergence from congeners. This discovery contributes to understanding the cryptic biodiversity of lumbricid earthworms in high‐altitude regions of southwestern China and underscores the importance of applying integrative taxonomic methods to resolve species boundaries in morphologically conserved groups.

Future research should focus on (1) whole‐genome resequencing to analyze hybrid zones and adaptive differentiation; (2) population genetic studies to assess gene flow and demographic history; and (3) establishment of an integrated species concept framework combining molecular, morphological, ecological, and geographic data to provide traceable and reproducible standard strains for ecotoxicological applications.

## Author Contributions

**Tingchang Liu:** data curation (equal), writing – original draft (equal), writing – review and editing (equal). **Rongchuan Xiong:** conceptualization (equal), project administration (equal), supervision (equal), writing – review and editing (equal). **Hong Chen:** data curation (equal), investigation (equal). **Jianhong Li:** investigation (equal). **Tingting Ji:** data curation (equal).

## Funding

This study was supported by the Technology Support Project of Liupanshui Science and Technology Bureau (Grant No. 52020‐2024‐0‐2‐17), the Key Laboratory of Earthworm Resource Development and Utilization in Liupanshui City (Grant No. 52020‐2024‐PT‐03) and Discipline Team Construction Project of Liupanshui Normal University (Grant No. LPSSY2023XKTD10).

## Conflicts of Interest

The authors declare no conflicts of interest.

## Data Availability

All the required data are uploaded as Supporting Information.
